# Belladonna, Mislabeled Extract of Dandelion

**Published:** 1848-08

**Authors:** 


*Belladonna, Mislabeled Extract of Dandelion.*—In our last number we gave an account of a case of poisoning by Belladonna purchased for the Extract of Dandelion. We have since learned that, several months ago, a similar accident occurred in the same way, another Druggist of this city having received from the Shakers a considerable quantity of the Extract of Belladonna purporting to be Dandelion. Since the publication of our last number, still another mistake has been made of the same description. The druggist who supplied the medicine which gave rise to the accident several months ago, informed us that he immediately reported the circumstance to the Mt. Lebanon Society, and was informed in reply that the Society were aware that a large quantity of the Belladonna had been incorrectly labeled, and sent over the United States. There seems to have been culpable negligence, not only in the wrong labeling, but in observing silence on the subject, after the mistake was known by those who had committed it. No efforts appear to have been made to apprize druggists, and the public, of the fact that they had sent abroad a large quantity of Belladonna labeled Dandelion. Is there no legal punishment for such abominable selfishness, and reckless disregard for human life? If not, there is a hiatus in the criminal law which should be filled as soon as possible. In the mean time, we hope the matter will be so extensively made known, that an effectual *quietus* will be put upon any future sales of medicines prepared by the *Shakers of Mt. Lebanon, N. Y.* The reader of the case reported in our last number, will have observed, that, in addition to the carelessness in confounding the two articles, the Belladonna Extract was a miserable preparation. Had it been a good article, the patient's life would probably have been sacrificed to the mistake.

